# Association between *aldehyde dehydrogenase 2* polymorphisms and the incidence of diabetic retinopathy among Japanese subjects with type 2 diabetes mellitus

**DOI:** 10.1186/1475-2840-12-132

**Published:** 2013-09-13

**Authors:** Kazunori Morita, Junji Saruwatari, Haruna Miyagawa, Yoshihiro Uchiyashiki, Kentaro Oniki, Misaki Sakata, Ayami Kajiwara, Akira Yoshida, Hideaki Jinnouchi, Kazuko Nakagawa

**Affiliations:** 1 Division of Pharmacology and Therapeutics, Graduate School of Pharmaceutical Sciences, Kumamoto University, Kumamoto, Japan; 2 Jinnouchi Clinic, Diabetes Care Center, Kumamoto, Japan; 3 Center for Clinical Pharmaceutical Sciences, Kumamoto University, Kumamoto, Japan

**Keywords:** Advanced glycation end products, Alcohol drinking, Aldehyde dehydrogenase 2, Diabetic retinopathy, Diabetic angiopathy, γ-glutamyltransferase, Oxidative stress, Type 2 diabetes mellitus

## Abstract

**Background:**

Mitochondrial aldehyde dehydrogenase 2 (ALDH2) detoxifies reactive aldehydes in the micro- and macrovasculature. These substrates, including methylglyoxal and 4-hydroxynonenal formed from glucose and lipids, cause protein carbonylation and mitochondrial dysfunction, forming advanced glycation end products (AGEs). The present study aimed to confirm the association between the inactive ALDH2*2 allele and diabetic retinopathy (DR).

**Methods:**

A retrospective longitudinal analysis was conducted, among 234 Japanese patients with type 2 diabetes mellitus (DM) (156 males and 78 females) who had no DR signs at baseline and were treated for more than half a year. The ALDH2*1/*2 alleles were determined using a real-time TaqMan allelic discrimination assay. Multivariate-adjusted hazard ratios (HRs) and 95% confidential intervals (CIs) for the cumulative incidence of the development of DR were examined using a Cox proportional hazard model, taking drinking habits and the serum γ-glutamyltransferase (GGT) level into consideration.

**Results:**

The frequency of the ALDH2*2 allele was 22.3%. Fifty-two subjects cumulatively developed DR during the follow-up period of 5.5 ± 2.5 years. The ALDH2*2 allele carriers had a significantly higher incidence of DR than the non-carriers (HR: 1.92; *P* = 0.02). The incidence of DR was significantly higher in the drinkers with the ALDH2*2 allele than in those with the ALDH2*1/*1 genotype (HR: 2.61; *P* = 0.03), while the incidence of DR in the non-drinkers did not differ significantly between the *ALDH2* genotype groups (*P* > 0.05). The incidence of DR was significantly higher in the ALDH2*2 allele carriers with a high GGT level than in the non-carriers with a high or low GGT level (HR: 2.45; *P* = 0.03; and HR: 2.63; *P* = 0.03, respectively).

**Conclusions:**

To the best of our knowledge, this is the first report of a significant association between the ALDH2*2 allele and the incidence of DR. These findings provide additional evidence that ALDH2 protects both microvasculature and macrovasculature against reactive aldehydes generated under conditions of sustained oxidative stress, although further investigations in larger cohorts are needed to verify the results.

## Introduction

Hyperglycemia promotes vascular damage in patients with diabetes mellitus (DM) via multiple mechanisms [1]. The presence of diabetic retinopathy (DR) is associated with an over two-fold higher risk of coronary events which increases with the progression of DR [2]. Advanced glycation end product (AGE) formation and oxidative stress in the mitochondria of the vasculature have been proposed to contribute to both micro- and macrovascular complications [1-3]. The mitochondrial aldehyde dehydrogenase 2 (ALDH2) expressed in the micro- and macrovasculature detoxifies the reactive aldehydes formed from glucose and lipids [4]. These substrates, including methylglyoxal, glyoxal, and 4-hydroxynonenal (4-HNE) cause protein carbonylation and mitochondrial dysfunction, forming AGEs [3-5].

A recent meta-analysis of genome-wide association studies identified a variant, rs671, in the *ALDH2* gene that was associated with variations in blood pressure (BP) in East Asians [6]. According to that analysis, the wild-type ALDH2*1 allele was identified as a risk factor for an elevated BP. Conversely, the allele was associated with a reduced risk of coronary artery disease (CAD). These associations are believed to be largely mediated by alcohol consumption, because this variant determines an individual’s tolerance to alcohol by altering the ALDH2 enzymatic activity [7]. Accordingly, the authors interpreted the deleterious effects of the ALDH2*1 allele on BP to be balanced by the protective effects of alcohol consumption on the lipid profile, thus resulting in a net reduction in the risk of CAD. We, however, have shown that alcohol consumption, even less than one drink/day, increases the risk of hypertension in Japanese individuals with the inactive ALDH2*2 allele [8].

Based on this information, the present study aimed to confirm that ALDH2 potentially protects the microvasculature and macrovasculature against reactive aldehydes and carbonyl stress, regardless of the etiology [1,3-9]. We therefore investigated the association between the inactive ALDH2*2 allele and the risk of DR among Japanese subjects with type 2 DM.

## Methods

A retrospective longitudinal analysis was conducted, among 234 Japanese patients with type 2 DM (156 males and 78 females) who had no DR signs at baseline and had been treated at the Jinnouchi Clinic, Diabetes Care Center in Kumamoto, Japan, for more than half a year between February 2002 and January 2011. The study protocol was approved by the institutional ethics committee, and written informed consent was obtained from each subject. The study was performed in accordance with the Declaration of Helsinki.

DR was diagnosed by a professional ophthalmologist using direct ophthalmoscopy or fundus fluorescein angiography. DR was staged as no retinopathy, nonproliferative diabetic retinopathy (NPDR) or proliferative diabetic retinopathy (PDR) according to the criteria determined at the third national ophthalmology conference held in 1985. The occurrence of DR was defined as having no DR signs in both eyes at baseline and developing NPDR or PDR in either of the eyes during the follow-up period.

Height and weight were measured using standard protocols, and the body mass index (BMI) was calculated. Fasting blood samples were analyzed using the standard methods of the Japan Society of Clinical Chemistry. The blood pressure (BP) was measured after the subject rested in a sitting position. Information regarding smoking and alcohol drinking habits was obtained via face-to-face interviews conducted by medical staff members. Based on their alcohol drinking habits, the subjects were categorized as non-drinkers (abstainers) or drinkers (including social drinkers). Additionally, the level of serum γ-glutamyltransferase (GGT) was measured and used as a biomarker for alcohol intake [10,11].

Genomic DNA was prepared from whole blood using a DNA purification kit (Flexi Gene DNA kit, QIAGEN, Hilden, Germany). The ALDH2*1/*2 alleles were determined using a real-time TaqMan allelic discrimination assay (Applied Biosystems, CA, USA) according to the protocols provided by the manufacturer (assay no. C_11703892_10). All reagents were purchased from Applied Biosystems. To ensure the genotyping quality, we included DNA samples as internal controls, hidden samples of known genotypes and negative controls (water).

The data are presented as the means ± standard deviations or proportions for categorical variables. Student’s *t*-test and Fisher’s exact test were used for comparisons of the continuous and categorical variables, respectively. Multivariate-adjusted hazard ratios (HRs) and 95% confidential intervals (CIs) for the cumulative incidence of DR were examined using a Cox proportional hazard model. In the multivariate model, we included the following variables as covariates: gender, BMI, diabetes duration, hemoglobin A1c (HbA1c), systolic BP and the GGT level. The interactive effect and combined effect between the *ALDH2* genotypes and the GGT levels at the baseline on the development of DR were also analyzed using a Cox proportional hazard model. Kaplan-Meier survival curves were used to estimate the DR-free survival according to the *ALDH2* genotypes or the combinations of the genotypes and the GGT levels. The GGT levels at the baseline were dichotomized according to the median values for males and females. Factors influencing the GGT level were determined using a univariate linear regression analysis with calculations of the β coefficient. The longitudinal relationship between the incidence of DR and the GGT values during the observation period was also assessed using an autoregressive model based on the generalized estimating equations approach. A *P* value of < 0.05 was considered to be statistically significant. All statistical analyses were performed using the SPSS software package (version 17.0, SPSS Inc., Chicago, IL).

## Results

The frequency of the ALDH2*2 allele was 22.3%, and that of the ALDH2*1/*1, *1/*2 and *2/*2 genotypes was 61.1%, 33.3% and 5.6%, respectively. The observed genotype frequency distribution was consistent with the Hardy-Weinberg equilibrium. Since the number of subjects with the ALDH2*2/*2 genotype was too small to assess the effects of the genotype on the development of DR, the ALDH2*1/*2 and ALDH2*2/*2 genotypes were combined in the subsequent analysis. The demographic characteristics of the subjects with the ALDH2*1/*1 genotype (non-carriers) and the ALDH2*2 allele carriers at baseline are shown in Additional file 1: Table S1. The frequency of drinkers and the mean GGT values were significantly lower in the ALDH2*2 allele carriers than in the non-carriers. The mean GGT value was significantly higher in the drinkers than in the non-drinkers (58.5 ± 70.0 IU/L vs. 34.6 ± 31.6 IU/L, *P* < 0.01). The median GGT value was 37 IU/L for the males and 26 IU/L for the females.

**Figure 1:**
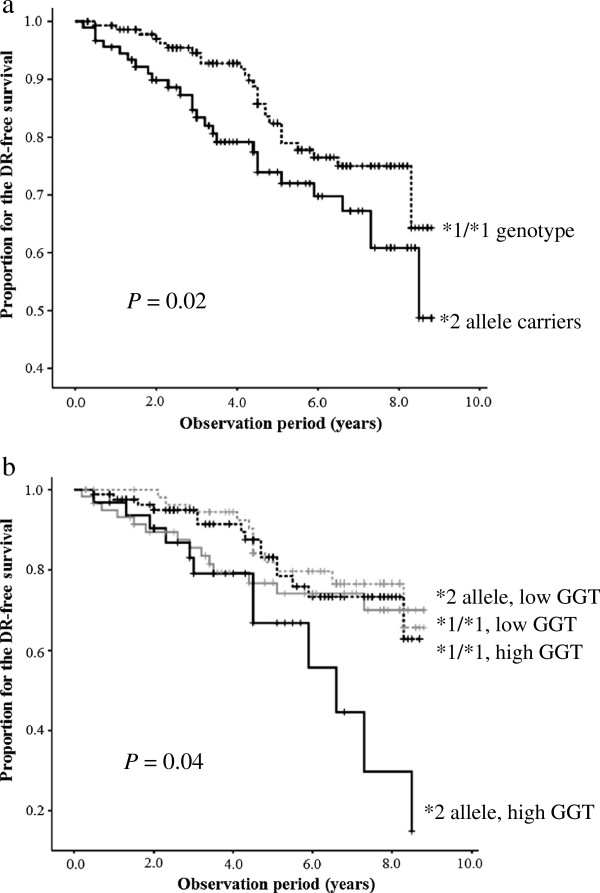
**Kaplan-Meier curves for diabetic retinopathy-free survival during the observation period.** The curves are described according to the ALDH2 genotype **(a)** and the combination of the ALDH2 genotype and the serum GGT level **(b)**. Adjusted for gender, body mass index, duration of diabetes, hemoglobin A1c and systolic blood pressure. ALDH2 aldehyde dehydrogenase 2, GGT γ-glutamyltransferase.

The mean follow-up duration was 5.5 ± 2.5 years. Fifty-two subjects cumulatively developed DR (51 cases of NPDR and one cases of PDR) during the follow-up period, and the incidence of DR was 58.6/1,000 person-years. The ALDH2*2 allele carriers exhibited a higher incidence of DR than the non-carriers (70.2/1,000 person-years and 50.7/1,000 person-years, respectively). A significantly higher incidence of DR among the ALDH2*2 allele carriers than the non-carriers was also observed in the Cox proportional hazard model (HR: 1.92; *P* = 0.02) (Additional file 1: Table S2, Figure 1a). In this model, the incidence of DR increased (HR: 1.26; *P* < 0.01) with increasing HbA1c (by 1.0%), but it was not influenced by gender, BMI, diabetes duration, systolic BP and high GGT level in all subjects (Additional file 1: Table S2). Among the ALDH2*2 allele carriers, the duration of diabetes was also added to the risk factors (HR: 1.11; *P* = 0.01) and a high GGT level appeared to be a potential risk factor (HR: 1.91; *P* = 0.14) for DR (Additional file 1: Table S2).

No interactive effects between the *ALDH2* genotypes and alcohol intake or the GGT levels were observed (*P* > 0.05). However, the incidence of DR was significantly higher in the drinkers with the ALDH2*2 allele than in those with the ALDH2*1/*1 genotype (HR: 2.61; *P* = 0.03), while the incidence of DR in the non-drinkers did not differ significantly between the ALDH2*2 allele carriers and non-carriers. Moreover, the incidence of DR in the ALDH2*2 allele carriers with a high GGT level was significantly higher than that observed in the non-carriers with a high or low GGT level (HR: 2.45; *P* = 0.03; and HR: 2.63; *P* = 0.03, respectively) (Table 1, Figure 1b).

**Table 1:** **Association between the risk of DR and the combination of the *ALDH2 *genotypes and the serum GGT levels**

**GGT levels**^**†**^	***ALDH2 *genotype**	**n**	**HR**^**‡ **^**(95% CI)**	***P*-value**	**HR**^**‡ **^**(95% CI)**	***P*-value**
Low	*1/*1 genotype	58	1	-	-	-
	*2 allele carrier	59	1.60 (0.77 - 4.09)	0.25	-	-
High	*1/*1 genotype	85	1.15 (0.53 - 2.49)	0.73	1	-
	*2 allele carrier	32	2.63 (1.12-6.18)	0.03	2.45 (1.10-5.48)	0.03

^†^GGT was dichotomized by the near-median 37 IU/L for the males and 26 IU/L for the females.

^‡^Adjusted by gender, BMI, duration of diabetes, HbA1c and systolic BP.

*DR*, diabetic retinopathy; *ALDH2*, aldehyde dehydrogenase 2; *GGT*, γ-glutamyltransferase; *HR*, hazard ratio; *CI*, confidence interval; *BMI*, body mass index; *HbA1c*, hemoglobin A1c; *BP*, blood pressure.

The demographic characteristics of the subjects stratified by the combination of the *ALDH2* genotypes and the GGT level at baseline are shown in Table 2. The mean values of BMI, BP and the levels of transaminases and the prevalence of hypertension were significantly higher in the subjects with a high GGT level than in those with a low GGT level, irrespective of the *ALDH2* genotype. Among the non-carriers, the frequency of drinkers was significantly higher in the subjects with a high GGT level than in those with a low GGT level, and a high GGT level was most significantly associated with drinking habits (Tables 2 and 3). Among the ALDH2*2 allele carriers, the frequency of drinkers was comparable between the subjects with high and low GGT levels, and a high GGT level was significantly associated with multiple factors, including an ever-smoking habit, hypertension, high systolic and diastolic BP and a high BMI.

**Table 2:** **Clinical characteristics of the subjects stratified by the combination of the *ALDH2 *genotype and the serum GGT level at baseline**

**GGT levels**^**†**^	**Low**		**High**		***P*-value**
***ALDH2 *genotype**	*1/*1	*2 allele	*1/*1	*2 allele	
	**(n = 58)**	**(n = 85)**	**(n = 59)**	**(n = 32)**	
Female (%)	37.9	28.8	31.8	37.5	0.69
Age (years)	57.6 ± 11.0	60.3 ± 9.7	57.5 ± 12.4	56.9 ± 11.0	0.40
Age of diagnosis (years)	47.5 ± 10.9	51.9 ± 11.1	49.4 ± 12.2	51.1 ± 9.2	0.18
Diabetes duration (years)	10.0 ± 8.7	8.3 ± 5.4	7.9 ± 7.0	6.1 ± 5.2	0.07
BMI (kg/m^2^)	23.2 ± 3.6	23.2 ± 2.9	24.8 ± 4.2	25.8 ± 4.0	< 0.01
Casual plasma glucose (mmol/L)	10.9 ± 4.6	11.4 ± 5.0	11.7 ±5.9	11.6 ± 3.4	0.85
HbA1c (%)	8.4 ± 2.1	8.5 ± 2.2	8.7 ± 2.1	9.2 ± 1.8	0.36
Systolic BP (mmHg)	133.5 ± 19.5	131.5 ± 20.8	141.7 ± 17.5	143.7 ± 20.7	< 0.01
Diastolic BP (mmHg)	80.9 ± 10.9	79.5 ± 12.3	86.2 ± 12.0	87.4 ± 11.8	< 0.01
Triglycerides (mmol/L)	1.3 ± 0.9	1.6 ± 1.5	2.6 ± 2.4	1.9 ± 0.8	< 0.01
HDL cholesterol (mmol/L)	1.6 ± 0.5	1.4 ± 0.4	1.4 ± 0.3	1.4 ± 0.3	0.08
LDL cholesterol (mmol/L)	3.1 ± 0.7	3.2 ± 0.7	3.1 ± 0.8	3.4 ± 0.8	0.21
AST (IU/L)	21.0 ± 5.1	21.6 ± 5.3	39.5 ± 24.4	37.7 ± 20.7	< 0.01
ALT (IU/L)	20.9 ± 9.2	20.7 ± 7.2	49.7 ± 37.8	50.3 ± 36.4	< 0.01
GGT (IU/L)	20.8 ± 7.9	21.4 ± 7.8	77.7 ± 78.7	60.6 ± 37.8	< 0.01
Hypertension (%)	44.8	42.4	64.7	68.8	< 0.01
Dyslipidemia (%)	56.9	64.4	75.3	71.9	0.12
Ever smoker (%)	48.2	43.1	54.2	50.0	0.64
Drinker (%)	56.9	30.5	72.9	28.1	< 0.01
Therapy components					
Hypoglycemic agents					
Oral hypoglycemic agents (%)	72.4	67.8	64.3	75.0	0.65
Insulin (%)	22.4	10.2	7.1	3.1	0.02
Antihypertensive agents					
ACE inhibitors or ARBs (%)	10.3	6.8	13.1	34.4	< 0.01
Others (%)	19.0	11.9	21.4	28.1	0.25
Agents for hyperlipidemia					
Fibrates (%)	1.7	1.7	2.4	0.0	1.00
Statins (%)	3.4	11.9	9.5	9.4	0.37
Others (%)	0.0	1.7	0.0	0.0	0.64

Data are percentages or mean ± standard deviation.

^†^GGT was dichotomized by the near-median 37 IU/L for the males and 26 IU/L for the females.

*ALDH2*, aldehyde dehydrogenase 2; *GGT*, γ-glutamyltransferase; *BMI*, body mass index; *HbA1c*, hemoglobin A1c; *BP*, blood pressure; *HDL*, high-density lipoprotein; *LDL*, low-density lipoprotein; *AST*, aspartate aminotransferase; *ALT*, alanine aminotransferase; *ACE*, angiotensin converting enzyme; *ARB*, angiotensin II receptor blocker.

**Table 3:** **Factors associated with the serum GGT level stratified by the *ALDH2 *genotype**

	ALDH2*1/*1 genotype			ALDH2*2 allele carrier		
	**β**	**SE**	***P *value**	**β**	**SE**	***P *value**
Age	−0.37	0.48	0.45	−0.70	0.30	0.02
Diabetes duration	−0.75	0.72	0.30	−1.38	0.57	0.02
BMI	0.52	1.39	0.71	1.80	0.87	0.04
Systolic BP	−0.22	0.30	0.94	0.29	0.14	0.046
Diastolic BP	0.50	0.47	0.30	0.62	0.24	0.01
Triglycerides	0.22	0.03	< 0.01	0.07	0.03	0.02
AST	1.48	0.24	< 0.01	0.97	0.18	< 0.01
ALT	0.85	0.16	< 0.01	0.56	0.10	< 0.01
Hypertension	1.10	11.33	0.92	13.34	6.12	0.03
Ever smokers	13.23	11.46	0.25	16.48	6.09	< 0.01
Drinkers	26.07	11.68	0.03	8.79	6.80	0.20
HbA1c	4.23	2.64	0.11	1.15	1.52	0.45

*GGT*, γ-glutamyltransferase; *ALDH2*, aldehyde dehydrogenase 2; *SE*, standard error; *BMI*, body mass index; *BP*, blood pressure; *AST*, aspartate aminotransferase; *ALT*, alanine aminotransferase; *HbA1c*, hemoglobin A1c.

The longitudinal changes in the GGT values during the observation period are shown in Table 4. The GGT values were significantly higher in the subjects with DR than in those without, and the difference was significant in the ALDH2*2 allele carriers (adjusted β: 12.30 IU/L; *P* = 0.02), but not in the non-carriers (adjusted β: 5.48 IU/L; *P* = 0.65). Only two subjects were newly identified to be drinkers, while the remaining subjects did not change their drinking habits during the observation period.

**Table 4:** **The change in the average GGT value stratified by the *ALDH2 *genotype**

	ALDH2*1/*1 genotype					ALDH2*2 allele carrier				
	**With the DR**		**Without the DR**			**With the DR**		**Without the DR**		
**Observation period**	**n**	**GGT (IU/L)**	**n**	**GGT (IU/L)**	***P* value**	**n**	**GGT (IU/L)**	**n**	**GGT (IU/L)**	***P* value**
0	26	66.1 ± 96.5	117	55.4 ± 76.2	0.54	26	42.7 ± 40.3	65	31.5 ± 24.2	0.11
2	22	63.6 ± 72.6	104	47.7 ± 62.7	0.30	23	37.7 ± 24.9	56	28.6 ± 18.3	0.08
4	20	47.3 ± 57.2	77	41.2 ± 44.6	0.61	20	38.2 ± 25.2	39	27.3 ± 16.9	0.05
6	16	61.4 ± 56.9	60	40.9 ± 45.5	0.20	16	31.6 ± 14.9	28	20.3 ± 7.7	< 0.01
8	10	59.4 ± 46.4	27	34.0 ± 19.5	0.12	14	36.4 ± 24.0	16	20.9 ± 8.4	0.02

Data are mean ± standard deviation.

*GGT*, γ-glutamyltransferase; *ALDH2*, aldehyde dehydrogenase 2; *DR*, diabetic retinopathy.

## Discussion

To the best of our knowledge, this is the first report of a significant association between the ALDH2*2 allele and the incidence of DR. In this retrospective longitudinal study of Japanese subjects with type 2 DM, an alcohol drinking habit, especially a high serum GGT level further increased the risk of DR in the ALDH2*2 allele carriers; however, these factors did not affect the risk in the non-carriers.

The allelic variant at rs671, ALDH2*2, is common in East Asians; however, it is very rare in other ethnic groups [4,6-8,12,13]. This point mutation alters the enzyme activity in a partially negative dominant fashion, and the variant homozygotes virtually lack any enzyme activity *in vivo* owing to its extremely high Km for coenzyme nicotinamide adenine dinucleotide (NAD^+^) and a much lower Vmax [12]. There is substantial evidence that the overproduction of reactive aldehydes such as methylglyoxal, glyoxal and 4-HNE results in protein carbonylation and activates multiple pathogenetically relevant pathways [9]. The structural and functional changes resulting from the increased formation of AGEs in the early metabolic environment are implicated in the development and progression of micro- and macrovascular complications in the long run, and are referred to as “metabolic memory” [3-5,14]. For instance, 4-HNA forms Michael adducts with dimethylarginine dimethylaminohydrolase 1 (DDAH1) and inactivate DDAH1, which is the major enzyme responsible for eliminating asymmetric dimethylarginine (ADMA) in the human endothelium [15]. Derangement of the L-arginine-nitric oxide pathway by ADMA has been implicated as an important factor contributing to endothelial dysfunction [15,16]. Therefore, elevated plasma levels of ADMA caused by the genetic or metabolic inactivation of DDAH1 have been reported to be associated with micro- and macrovascular diabetic complications [15,16]. ALDH2 detoxifies the reactive aldehydes in the vasculatures. Consequently ALDH2 dysfunction can result in the accelerated accumulation of aldehydes and the development of vascular complications, such as DR [4-6,8,9].

ALDH2*2 allele carriers exhibit a marked rise in the level of acetaldehyde in the blood and develop acute cardiovascular reactions, such as facial flushing, tachycardia and orthostatic hypotension, following alcohol consumption [12]. Consequently, the ALDH2*2 allele is the strongest genetic modifier of drinking behavior and reduces the risk of alcohol-related diseases, such as alcoholism, alcohol-related liver cirrhosis, chronic pancreatitis, DM and hypertension [5-8,12,17]. Past studies [18,19] and this study have shown that alcohol intake is not an independent risk factor for DR. In this study, however, the ALDH2*2 allele was a significant risk for DR only in drinkers. Light to moderate alcohol intake is known to reduce the incidence of macrovascular diseases in a U-shaped fashion [20]. Ethanol at low concentrations is metabolized by low Km alcohol dehydrogenase and ALDH2, thus resulting in the production of reduced NAD^+^[21]. These reactions create a reductive environment, decrease oxidative stress and prevent the secondary production of aldehydes via lipid peroxidation. Therefore, chronic low ethanol intake confers a beneficial effect on the vasculature primarily through its ability to increase the antioxidant capacity and lower the amount of AGEs [20,21]. Conversely, the lack of ALDH2 activity increases the levels of acetaldehydes and other reactive aldehydes, and thus induces oxidative stress even after light alcohol intake [21]. Previously, we reported that alcohol intake increases the risk of hypertension and elevates systolic and diastolic BP much more significantly in ALDH2*2 allele carriers than in non-carriers in a dose-dependent manner [8]. Taken together, the ALDH2*2 allele is thus considered to be an independent genetic risk factor for alcohol-related microvascular and macrovascular diseases [4,6,8]. One study showed that alcohol intake was associated with a lower risk of all microvascular complications in a U-shaped fashion among Type 1 diabetes patients in Europe, where the ALDH2*2 allele is very rare [22]. The present study also showed drinkers with the ALDH2*1/*1 genotype tended to be at a lower risk for the incidence of DR than non-drinkers (data not shown). These facts suggest that ALDH2 protects the vasculature from reactive aldehydes in relation to the beneficial effects of mild to moderate alcohol intake [6,8,20-22].

We further investigated the data after taking into account the GGT level, a valid marker of excessive alcohol consumption [10,23] The occurrence of DR was significantly higher in the ALDH2*2 allele carriers with a high GGT level at baseline than in the non-carriers with a high or low GGT level (Table 1, Figure 1b). The GGT levels in non-carriers should be dependent on alcohol intake, because the frequency of drinkers was significantly higher among the non-carriers with a high GGT level (Table 2), and a high GGT level was most significantly associated with drinking habits (Table 3). Among the ALDH2*2 allele carriers, the frequency of drinkers was comparable between the subjects with high and low GGT levels (Table 2), and a high GGT level was significantly associated with multiple cardiovascular risk factors, such as an ever-smoking habit, hypertension, high systolic and diastolic BP and a high BMI (Table 3). GGT metabolizes extracellular glutathione (GSH), allowing precursor amino acids to be reused for intracellular GSH synthesis; hence, a modest increase within the normal range may be an early marker of oxidative stress [10]. Positive associations have been reported between the serum GGT level and the incidence of type 2 DM, all-cause and cardiovascular disease death in DM patients, diabetic nephropathy and DR [11,23-26]. In this study, the GGT values during the observation period were significantly higher in the subjects with DR than in those without among the ALDH2*2 allele carriers (Table 4). The pathophysiological mechanisms underlying these associations have not been elucidated; however, insulin resistance, oxidative stress and chronic low-grade systemic inflammation may be involved [10,11], and these pathological conditions were also reported in the ALDH2*2 carriers at an early stage of glucose intolerance [27]. When our present findings and these previous findings are combined, it appears that a high GGT level in ALDH2*2 carriers may have some predictive value for DR.

The crucial limitations of the present study include the retrospective study design and small sample size. In addition, the details of the subjects’ drinking habits, the influence of drug therapy were uncertain.

In conclusion, we herein demonstrated that the ALDH2*2 allele is an independent risk factor for the incidence of DR. The association between the *ALDH2* genotype and the incidence of DR was significant among drinkers and subjects with a high GGT level. These findings provide additional evidence that ALDH2 protects both the microvasculature and macrovasculature against the reactive aldehydes generated under conditions of sustained oxidative stress, although further investigations in larger cohorts are needed to verify the results.

## Abbreviations

ACE: Angiotensin converting enzyme; ADMA: Asymmetric dimethylarginine; AGE: Advanced glycation end product; ALDH2: Aldehyde dehydrogenase 2; ALT: Alanine aminotransferase; ARB: Angiotensin II receptor blocker; AST: Aspartate aminotransferase; BMI: Body mass index; BP: Blood pressure; CAD: Coronary artery disease; CI: Confidence interval; DDAH1: Dimethylarginine dimethylaminohydrolase 1; DM: Diabetes mellitus; DR: Diabetic retinopathy; GGT: γ-glutamyltransferase; GSH: Glutathione; HbA1c: Hemoglobin A1c; HDL: High-density lipoprotein; 4-HNE: 4-hydroxynonenal; HR: Hazard ratio; LDL: Low-density lipoprotein; NAD: Nicotinamide adenine dinucleotide; NPDR: Nonproliferative diabetic retinopathy; PDR: Proliferative diabetic retinopathy.

## Competing interests

The authors declare that they have no competing interests.

## Authors’ contributions

All authors have made substantial contributions to conception and design, or acquisition of data, or analysis and interpretation of data; have been involved in drafting the manuscript or revising it critically for important intellectual content; and have given final approval of version to be published. All authors read and approved the final manuscript.

## Supplementary Material

Additional file 1: Table S1 Clinical characteristics of subjects stratified by the *ALDH2* genotype at the baseline. **Table S2**. Association between the risk for DR and the covariates. **Table S3**. Clinical characteristics of subjects stratified by drinking habit at the baseline. Click here for file
