# Supplementary material for: Association between aldehyde dehydrogenase 2 polymorphisms and the incidence of diabetic retinopathy among Japanese subjects with type 2 diabetes mellitus
Source: Cardiovasc Diabetol. 2013 Sep 13;12:132. doi: 10.1186/1475-2840-12-132 (PMC3847457; doi:10.1186/1475-2840-12-132)
Supplement: Additional file 1: Table S1 — Clinical characteristics of subjects stratified by the ALDH2 genotype at the baseline. Table S2. Association between the risk for DR and the covariates. Table S3. Clinical characteristics of subjects stratified by drinking habit at the baseline. [file 1475-2840-12-132-S1.docx]

Table S1. Clinical characteristics of the subjects stratified by the *ALDH2* genotype at the baseline.

|  | All subjects  (n = 234) |  | **1/*1* genotype  (n = 143) | **2* allele carrier  (n = 91) | *P*-value |
| --- | --- | --- | --- | --- | --- |
| Female (%) | 33.3 |  | 34.3 | 31.9 | 0.78 |
| Age (years) | 58.2 ± 11.2 |  | 57.5 ± 11.8 | 59.1 ± 10.2 | 0.29 |
| Age of diagnosis (years) | 49.8 ± 11.3 |  | 48.7 ± 11.7 | 51.6 ± 10.4 | 0.05 |
| Diabetes duration (years) | 8.3 ± 7.0 |  | 8.7 ± 7.8 | 7.5 ± 5.4 | 0.16 |
| BMI (kg/m^2^) | 24.1 ± 3.8 |  | 24.1 ± 4.0 | 24.1 ± 3.5 | 0.96 |
| Fasting plasma glucose (mmol/L) | 11.4 ± 5.0 |  | 11.4 ± 5.4 | 11.5 ± 4.4 | 0.86 |
| HbA1c (%) | 8.6 ± 2.1 |  | 8.5 ± 2.1 | 8.7 ± 2.1 | 0.48 |
| Systolic BP (mmHg) | 137.4 ± 19.8 |  | 138.4 ± 18.7 | 135.8 ± 21.5 | 0.33 |
| Diastolic BP (mmHg) | 83.4 ± 12.2 |  | 84.1 ± 11.8 | 82.3 ± 12.6 | 0.28 |
| Triglycerides (mmol/L) | 1.9 ± 1.8 |  | 2.1 ± 2.0 | 1.7 ± 1.3 | 0.11 |
| HDL cholesterol (mmol/L) | 1.4 ± 0.4 |  | 1.5 ± 0.4 | 1.4 ± 0.3 | 0.40 |
| LDL cholesterol (mmol/L) | 3.1 ± 0.8 |  | 3.1 ± 0.8 | 3.2 ± 0.8 | 0.12 |
| AST (IU/L) | 30.2 ± 19.1 |  | 32.0 ± 21.1 | 27.2 ± 15.0 | 0.06 |
| ALT (IU/L) | 35.3 ± 30.6 |  | 38.0 ± 32.9 | 31.1 ± 26.3 | 0.09 |
| GGT (IU/L) | 47.1 ± 56.2 |  | 54.6 ± 66.9 | 35.2 ± 29.8 | < 0.01 |
| Hypertension (%) | 54.7 |  | 56.6 | 51.6 | 0.50 |
| Dyslipidemia (%) | 67.5 |  | 67.8 | 67.0 | 1.00 |
| Ever smoker (%) | 49.3 |  | 51.8 | 45.6 | 0.42 |
| Drinker (%) | 52.1 |  | 66.4 | 29.7 | < 0.01 |
| Therapy components |  |  |  |  |  |
| Hypoglycemic agents |  |  |  |  |  |
| Oral hypoglycemic agents (%) | 68.7 |  | 67.6 | 70.3 | 0.77 |
| Insulin (%) | 11.2 |  | 13.4 | 7.7 | 0.21 |
| Antihypertensive agents |  |  |  |  |  |
| ACE inhibitors or ARBs(%) | 13.7 |  | 12.0 | 16.5 | 0.34 |
| Others(%) | 19.3 |  | 20.4 | 17.6 | 0.62 |
| Agents for hyperlipidemia |  |  |  |  |  |
| Fibrates(%) | 1.7 |  | 2.1 | 1.1 | 1.00 |
| Statins(%) | 8.6 |  | 7.0 | 11.0 | 0.34 |
| Others (%) | 0.4 |  | 0.0 | 1.1 | 0.39 |

Data are percentages or mean ± standard deviation

ALDH2: aldehyde dehydrogenase 2; BMI: body mass index; HbA1c: hemoglobin A1c; BP: blood pressure; HDL: high-density lipoprotein; LDL: low-density lipoprotein; AST: aspartate aminotransferase; ALT: alanine aminotransferase; GGT: γ-glutamyltransferase; ACE: angiotensin converting enzyme; ARB: angiotensin II receptor blocker.

Table S2. Association between the risk of DR and the covariates.

|  | All subjects | |  | **1/*1* genotype | |  | **2* allele carrier | |
| --- | --- | --- | --- | --- | --- | --- | --- | --- |
|  | HR (95% CI) | *P*-value |  | HR (95% CI) | *P*-value |  | HR (95% CI) | *P*-value |
| *ALDH2* **2* allele carrier | 1.92 (1.09 - 3.39) | 0.02 |  | - | - |  | - | - |
| HbA1c | 1.26 (1.11 - 1.43) | < 0.01 |  | 1.31 (1.09 - 1.56) | < 0.01 |  | 1.26 (1.02 - 1.56) | 0.03 |
| Diabetes duration | 1.04 (1.00 - 1.09) | 0.05 |  | 1.02 (0.96 - 1.07) | 0.59 |  | 1.11 (1.02 - 1.20) | 0.01 |
| Systolic BP | 1.01 (1.00 - 1.02) | 0.11 |  | 1.02 (1.00 - 1.05) | 0.10 |  | 1.01 (0.99 - 1.03) | 0.42 |
| Female | 1.70 (0.96 - 3.00) | 0.07 |  | 1.25 (0.54 - 2.88) | 0.60 |  | 1.98 (0.99 - 4.51) | 0.11 |
| BMI ≥ 25 | 1.03 (0.57 - 1.87) | 0.93 |  | 0.88 (0.35 - 2.21) | 0.78 |  | 1.15 (0.49 - 2.70) | 0.76 |
| High GGT^†^ | 1.37 (0.77 - 2.43) | 0.28 |  | 1.05 (0.47 - 2.33) | 0.91 |  | 1.91 (0.82 - 4.49) | 0.14 |

^†^GGT was dichotomized by the near-median 37 IU/L for the males and 26 IU/L for the females.

DR: diabetic retinopathy; HR: hazard ratio; CI: confidence interval; ALDH2: aldehyde dehydrogenase 2; HbA1c: hemoglobin A1c; BP: blood pressure; BMI: body mass index; GGT: γ-glutamyltransferase.

Table S3. Clinical characteristics of subjects stratified by drinking habit at the baseline.

|  | Drinkers  (n = 122) | Non-drinkers  (n = 112) | *P*-value |
| --- | --- | --- | --- |
| Female (%) | 13.1 | 55.4 | < 0.01 |
| Age (years) | 58.0 ± 9.9 | 58.2 ± 12.6 | 0.84 |
| Age of diagnosis (years) | 48.6 ± 10.4 | 51.1 ± 12.1 | 0.10 |
| Diabetes duration (years) | 9.3 ± 7.8 | 7.1 ± 5.7 | 0.01 |
| BMI (kg/m^2^) | 24.0 ± 3.4 | 24.3 ± 4.3 | 0.46 |
| Casual plasma glucose (mmol/L) | 11.7 ± 5.5 | 11.1 ± 4.6 | 0.44 |
| HbA1c (%) | 8.5 ± 2.1 | 8.8 ± 2.1 | 0.29 |
| Systolic BP (mmHg) | 136.9 ± 18.8 | 137.8 ± 20.9 | 0.73 |
| Diastolic BP (mmHg) | 83.5 ± 12.2 | 83.3 ± 12.2 | 0.89 |
| Triglycerides (mmol/L) | 2.2 ± 2.2 | 1.6 ± 1.2 | 0.01 |
| HDL cholesterol (mmol/L) | 1.5 ± 0.4 | 1.4 ± 0.4 | 0.43 |
| LDL cholesterol (mmol/L) | 3.1 ± 0.8 | 3.2 ± 0.7 | 0.09 |
| AST (IU/L) | 30.9 ± 20.9 | 29.4 ± 16.9 | 0.53 |
| ALT (IU/L) | 35.8 ± 32.3 | 34.9 ± 28.8 | 0.82 |
| GGT (IU/L) | 58.5 ± 70.0 | 34.6 ± 31.6 | < 0.01 |
| Hypertension (%) | 53.3 | 56.3 | 0.69 |
| Dyslipidemia (%) | 69.7 | 65.2 | 0.49 |
| Ever smoker (%) | 66.7 | 30.3 | < 0.01 |
| Therapy components |  |  |  |
| Hypoglycemic agents |  |  |  |
| Oral hypoglycemic agents (%) | 64.5 | 73.2 | 0.16 |
| Insulin (%) | 10.7 | 11.6 | 0.84 |
| Antihypertensive agents |  |  |  |
| ACE inhibitors or ARBs(%) | 11.6 | 16.1 | 0.35 |
| Others(%) | 17.4 | 21.4 | 0.51 |
| Agents for hyperlipidemia |  |  |  |
| Fibrates(%) | 2.5 | 0.9 | 0.62 |
| Statins(%) | 5.0 | 12.5 | 0.06 |
| Others (%) | 0.8 | 0.0 | 1.00 |

Data are percentages or mean ± standard deviation

BMI: body mass index; HbA1c: hemoglobin A1c; BP: blood pressure; HDL: high-density lipoprotein; LDL: low-density lipoprotein; AST: aspartate aminotransferase; ALT: alanine aminotransferase; GGT: γ-glutamyltransferase; ACE: angiotensin converting enzyme; ARB: angiotensin II receptor blocker.
